# Proteomics Advances in the Understanding of Pollen–Pistil Interactions

**DOI:** 10.3390/proteomes2040468

**Published:** 2014-09-29

**Authors:** Ziyang Fu, Pingfang Yang

**Affiliations:** Key Laboratory of Plant Germplasm Enhancement and Speciality Agriculture, Wuhan Botanical Garden, Chinese Academy of Sciences, Wuhan 430074, China; E-Mail: ziyangrd@163.com

**Keywords:** proteomics, interaction, pollen, pistil, self-incompatibility

## Abstract

The first key point to the successful pollination and fertilization in plants is the pollen-pistil interaction, referring to the cellular and molecular levels, which mainly involve the haploid pollen and the diploid pistil. The process is defined as “siphonogamy”, which starts from the capture of pollen by the epidermis of stigma and ends up with the fusion of sperm with egg. So far, the studies of the pollen-pistil interaction have been explicated around the self-compatibility and self-incompatibility (SI) process in different species from the molecular genetics and biochemistry to cellular and signal levels, especially the mechanism of SI system. Among them, numerous proteomics studies based on the advanced technologies from gel-system to gel-free system were conducted, focusing on the interaction, in order to uncover the mechanism of the process. The current review mainly focuses on the recent developments in proteomics of pollen-pistil interaction from two aspects: self-incompatible and compatible pollination. It might provide a comprehensive insight on the proteins that were involved in the regulation of pollen-pistil interaction.

## 1. Introduction

Deep in the evolutionary history, the plant kingdom goes through trends from low to high, simple to complex, aquatic to terrestrial. Corresponding to the life cycles, it can be of three-types of propagation: vegetative propagation, asexual reproduction, and sexual reproduction. The sexual reproduction is independent of water during fertilization. The transporting of the sperm to the egg is via the pollen tube. Angiosperms are the largest and the most diverse groups in the plant kingdom. They are placed on top of the evolution table, showing alternation of generation in their life cycle. Their sporophyte produce spores meiotically in their asexual reproduction [1]. Angiosperms sexual reproduction occurs when the female and male gametes fuse during the process of fertilization to produce viable offspring. The formation of a viable zygote in angiosperms is dependent on successful pollination and fertilization, which begins with pollen grain landing on the stigma of the pistil. The first key point of this complex process of fertilization is the pollen-pistil interaction, referring to a cellular and molecular interaction where the haploid pollen and the diploid pistil fuse. The specific steps of the interaction are as follows: before the substantive contact, the immobile pollen is transferred by external environmental forces, such as wind or insects. Upon pollen grain landing on the surface of stigma, the recognition starts at molecular and cellular levels. The “compatible” pollen germinates if the surrounding conditions are suitable. Then, the pollen tube grows and extends through the style into the ovule; the sperm cells are discharged by female gametes. The series of process is also defined as “siphonogamy” [2]. It has significant role in reproductive biology of flowering plants. This kind of interaction is vital in sexual reproduction in order to obtain genetic diversity in a population. In addition, the interaction between the pollen and pistil is regarded as a series of stages while studying the molecular mechanisms underlying cell recognition, cell germination and cell-to-cell communication. Scrutinizing the mechanisms of interaction during fertilization will be beneficial to crop breeding and reproduction. So far, the studies of the pollen-pistil interaction have been explicated around the self-compatibility and self-incompatibility process in different species from the molecular genetics and biochemistry to cellular and signal level, especially the generation mechanism of SI system [3,4,5].

Thanks to the completed genome sequences of some model plants, such as Arabidopsis, the genus is Populus and rice, and the field has been expanded into genomics and proteomics [6]. Among them, proteomics, as the study of how proteins work, interact, diversify and specialize on a global scale, has been widely applied in analyzing the biological processes drawing support from the rapid development of mass spectrometry (MS). By these new and powerful proteomic techniques, the interaction between pollen and pistil can be improved and studied with new insight [7]. Looking back into the history of its development, the techniques in proteomic research can be divided into two categories: gel-based strategy, developed from 2-D gel electrophoresis (2-DE) and isoelectric focusing PAGT (IEF-PAGE) to differential in-gel electrophoresis (DIGE), and gel-free system based on the liquid chromatography-mass spectrometry (LC-MS), which analyzes proteins on a large-scale [8,9]. Among them, the differential in-gel electrophoresis (DIGE) uses different fluorescent dyes on the 2D-GE to enhance the accuracy of quantifying the expression level of different proteins. Additionally, LC-MS/MS-based quantitative methods can be improved with the different labeling strategies, such as isobaric tag for relative and absolute quantitation (iTRAQ) [10]. Recently, a new technique label-free quantification has been improved, and it is termed “label-free LC MS/MS” [11].

This review focuses on the advance of proteomics studies in the interaction between pollen and pistil to reveal the complex biological process from a pivotal gene or enzyme to an integral pathway of regulatory or signal transduction. 

## 2. Proteomic Analysis of Pollen-Pistil Interaction with Successful Fertilization

### 2.1. Proteome Dynamics in Pollen at Different Points from Development to Germination

Before cell-to-cell interaction, pollen has to undergo a complex and necessary development to form a suited grain with high quality. Anther with four locules is the progenitor cells to release microspores, which is generated from microsporocyte via two continuous meiotic divisions [12]. The microspores released in the locule undergo cytoplasmic reorganization mediated by the cytoskeleton, and it also termed as polarizability. Subsequently, the polarized cell forms a male germ unit not only with two sperm cells that fuse with egg cell and central cell, respectively, but also with a vegetative nucleus via two mitotic divisions. The pollen with the three cells is called mature pollen, which can interact with gynoecia and be selected into the style for tube growth to access the embryo sac for double fertilization [13,14]. Mature pollen germinates fast after pollination from quiescent to active state, and the vegetative cell grows a pollen tube transporting the sperm cells into the embryo sac to complete the double fertilization process. 

According to previous study on the pollen transcriptome in the model species *Arabidopsis*, 992 pollen-expressed mRNAs were identified by comparing with that of the sporophyte. Among them, nearly 40% were detected specifically in mature pollen, encoding proteins involved in carbohydrate metabolism and cytoskeleton dynamics, which would be a key to ensure the quality of pollen through regulatory and functional specialization [15]. Furthermore, one-third of the genes constitutively expressed in the vegetative tissues were not expressed in pollen. These results revealed that the transcriptome of gamete showed more simplicity and higher proportion of selectively expressed genes than sporophytic tissues [16]. Soon afterwards, a proteome map for mature pollen of *Arabidopsis thaliana* was drawn using 2-DE followed by ESI-MS/MS to expound the biochemistry of pollen [17]. Among the 135 identified proteins, there were almost 20% involved in metabolism, 17% in energy generation, or 12% in cell structure, which is similar to the study at transcriptional level. Moreover, seven proteins whose RNAs were not shown in the transcriptome have functions in metabolism, energy generation or cell structure. To resolve the omission of short and low abundant proteins, a new generic deterministic peptide classification scheme was set up to identify the proteins with minimized error rate on the Arabidopsis pollen [18]. In addition to the large scale on the pollen grains, the analysis on the pollen coat at gene level and protein levels had been taken [19]. There were 322 special proteins in mature pollen of rice using MS technologies, which had been classified into at least 14 functional categories [20]. Among them, 38 unique proteins were beta-1,4-xylanase and beta-glucanase in pollen coats, which were major proteins in the pollen coat of maize [21]. The released proteins from the pollen might contribute to the germination and growth of the grains and pollen tube. 

In a way, the revelation of the gene level cannot directly link to the discipline of the protein level and activity. The proteomics work on developmental pollen used two-dimensional gel to find disparate points and then identified them by MALDI-TOF MS based on the sequence dates. For example, in *Oryza sativa*, the comparative proteins of the different stages of pollen (pollen mother cell, tetrad, early young microspore, middle young microspore, early binucleate, late binucleate, heading stage) were taken into analysis by similar proteomics technologies. The 33 unique proteins with the same changing trend participated in sugar metabolism, cell elongation and expansion, which were essential to the pollen germination [22]. Analogously, rice mature (MPG) and germinated (GPG) pollen grains were selected artificially and then flowed to 2D-gels to obtain protein spots. Comparing proteins of the two different growth stages, 186 proteins from almost 2300 proteins were differentially expressed. These proteins are involved in regulatory and metabolic processes, such as the dynamics of protein and cytoskeleton [23]. Comparing the protein expression of the germinating pollen (GP) and the mature pollen (MP) of canola (*Brassica napus*) via DIGE associated to MALDI-TOF/TOF, the up-regulated proteins play roles in carbohydrate, nucleotide and protein metabolism, signal transduction and stress responses. On the contrary, some catalases and LEA proteins were showed to be down-regulated. These showed that the proteins associated in macromolecules’ metabolism and enzymes involved in the signal pathways were essential to the pollen germination [24]. The differentially expressed proteins in pollen tubes compared to the un-germinated pollens could demonstrate the special physiological processes that occur during the development of the pollen tubes [25]. Recently, a proteomics analysis was also conducted in pollen (before-pollinated and/or pollinated) and pollen tube of the *Picea meyer* [26], *Triticosecale wittmack* [27] and *Lilium davidii* [28]. These researches are better conducted in two aspects: pollen collection, including the condition of pollen grains germination *in vitro*, the nutrition and temperature for plants growth, pollen review by light microscopy, and the identification and quantification of proteomics, covering the ITRAQ labeling method and MALDI analysis by TOF/TOF instrument. The proteomics for male gametophyte contribute a new slight on examining the molecular mechanism and lay a great foundation for the study of fertilization. 

### 2.2. Protein Analysis on Pistils by Comparative Proteomics

Pistil, another participant, is composed of stigma, style, and ovary [29]. The apical stigma is in the position of capturing and ingesting pollen grains. The style can be regarded as a subtle bridge linking the stigma and the ovary, where the transmitting tissue plays role accompanying the extension of the pollen tube. The basal ovary is an organ containing the ovules, which gestate the embryo sac to grow the eggs [30,31]. As the name suggests, pollination depends on transferring male gametophyte (pollen grain) from the anther to the sigma of pistil. That is the space where interaction occurs. Judging by whether or not there is secretion on the surface, the stigmas can be classified into two broad categories, wet and dry. Comparing the two biochemical reactions based on the different structure, up to date, pollen capture by the wet stigmatic secretion is nonspecific while the recognition process shows a degree of species specificity in the dry-type stigma. Besides, pollen hydration within the secretion is passive and unregulated. On the contrary, it is a regulated process in dry stigma [2].

Crucifer is the typical family with dry stigma, which is selected as model plants for study of pollen-pistil interaction. Through comparing the whole-genome transcriptional profiles of stigmas and ovaries isolated separately from wild-type *Arabidopsis* and transgenic plants, in which cells of the stigma epidermis and transmitting tract were ablated by expression of a cellular toxin on the microarray platform, 115 and 34 genes were identified from 23,000 genes on the array to be expressed specifically in the stigma epidermis and transmitting tract. The proteins encoded by these genes were functional classified in signal transduction pathway, regulating the components of the extracellular matrix during pollination. Among them, S-locus receptor kinase (SRK), M-locus protein kinase (MLPK) and arm repeat containing (ARC1) play roles in self-incompatibility [32].

The affymetrix ATH1 whole genome array were used in comparing the different gene levels in un-pollinated pistils and un-fertilized ovules of *Arabidopsis thaliana*, as well as the pollinated pistils in special timing points that represented the most significant development from pollination to fertilization. The result showed 1373 genes were differentially expressed during pollen–pistil interaction, whose function were explained and projected to the extent necessary for successful fertilization [33]. The temporal and spatial gene expression profile of *in vivo* pollen-pistil interaction provided a detailed evidence of changing in gene expression pattern, further supporting the molecular mechanisms operating during pollination. 

Recent researches on proteomic analysis of the pistil in crucifer were mainly taken in SI response to find the candidate proteins. This implies that the mechanism of compatible interaction between pollen and pistil is not still directly addressed, especially the proteome in pistil. After searching—a comparative proteomic analysis of pistil before and after pollination in Soybean cooperated with the proteome database [34] and transcriptomics analyses [35]—a strict self-pollination plant was found. According to the MALDI-TOF-MS results based on the 2D-gel, 58 differently expressed proteins were identified, of which there were 22 up-regulated proteins and 36 down-regulated proteins after pollination. After functional classification, the largest group was metabolism-related proteins. Among them, the sucrose-phosphate synthase (U18) was increased in expression, while some isoforms of glutamine synthetase were decreased. These indicated that the primary metabolisms were enhanced to facilitate the pollination and the following pollen tube growth [36]. This study enhances our understanding of the level of proteins expression, as well as the participated biological processes. 

The representative plants with the wet stigma are mainly Solanaceae, Rosaceae and Liliaceae [2]. The stigmatic secretion (SE) on the surface of the stigma plays an important role in ingesting the compatible pollen grains. Except the lipid and carbohydrate, SE also contains a wide range of proteins with profound functions, such as the sigma-specific protein 1 (STIG1), regulating the timing of the accumulation of SE in tobacco and petunia [37]. The first analysis of SE proteins on a large-scale level was taken in the *Lilium longiflorum* and *Olea europaea* by SDS-PAGE coordinated with the LC-MS/MS. However, given the un-completely genome annotation of the two plants, a database search algorithm (Mascot) was employed and identified 51 proteins in Lily and 57 in olive, of which only 13 were present in both SEs. In-depth analysis of these proteins showed that more than half of the proteins contain a signal peptide, and it was predicted that the SE might participate in at least 80 different biological processes and 97 molecular functions, of which included the carbohydrate metabolism, cell signaling and response to the biotic and abiotic stresses. During pollination, the catabolic enzymes disintegrate large polysaccharides and lipids into smaller units to regulate pollen tube growth by selective degradation of cell-wall polysaccharides. Two Stigma/stylar cysteine-rich adhesion (SCA) isoforms, a chemotropic protein and a fasciclin-like domain (FAS) protein were identified in the lily SE with the role of pollen tube adhesion [38]. By proteomics, a comprehensive map of proteins can be built to discover their biological function within pollination.

When the male and female gametes are prepared for the next journey, the recognition and interaction will induce the tube growth via compatible signal and chemical gradients. The extension keeps until the tube enters into the embryo sac, where the molecules and receptors of tube response with the guidance from the female gametophyte [39]. Comparing to angiosperm, the pollination droplet is a unique and conservative pollination mechanism in gymnosperm. Proteomics analysis and identification taken on the mechanism revealed the proteins participating in the drop were related to the pathogen defense and pollen development [40]. Although there have not been a complete network for the interaction between the tube and pistil, some key regulators and receptors have been identified by multidisciplinary approaches including biochemistry, molecular genetics and functional genomics. On the contrary, in *Arabidopsis*, the female gametophytic guidance is divided into two stages: the funicular guidance, implying that the tubes extend from the septum to the funiculus, and the micropylar guidance, implying that the tube navigates from the funiculus to the female gametophyte through the micropyle [41]. For example, MYB98, as a transcription factor, expresses in the synergid cell as micropyle guidance [42,43]. AtLURE1 peptides belong to the defensin-like (DEFL) peptides, expressed in synergid cells and secreted toward the funicular surface to guide the tube growth as especially guidance [44]. Looking back on how the receptors on the pollen tube interact with the female guidance, the ion gradient regulated by the transmembrane proteins and channels are critical for the tube growth, such as the cyclic nucleotide-gated channel 18 (CNGC18), working as a channel to regulate the Ca^2+^ concentration, and a GABA transaminase, which is coded by the *pollen pistil 2* (*POP2*) and has the function to increase content of GABA in the style and ovary [39,45]. Besides, lost in pollen tube guidance 1 (LIP1) and 2 (LIP2) were verified as a receptor complex to respond to AtLURE1 [46]. Especially, the mitogen-activated protein kinase 3 (MPK3) and the mitogen-activated protein kinase 6 (MPK6) were verified with the function of guiding the direction to the right towards the mutational plants of mpk3 and mpk4 [47].

Although much research had been conducted on several model species around compatible and even incompatible pollination and fertilization, there was a common theory among the types of molecular regulation on the pollen-pistil interaction [48]. Researchers suggested the absence was as a result of rapid evolution and change of proteins regulating sexual reproductive process [49]. For instance, arabinogalactan proteins (AGPs), a family of hydroxyproline-rich glycoproteins (HRGPs), showed an increased expression during pollination in the olive pistil (*Olea europaea* L.) compared to the non-pollinated pistil, while the expression of AGPs decreased after pollination. AGPs were localized predominantly in the cell wall of secretory cell of the stigma, as well as in the transmitting tissue of the pistil during the pollination period by means of immunofluorescence localization. These results proved that proteins play roles of supporting pollen performance and tube growth during the pollination stage in olive, which corresponds to the conclusion that AGPs had roles in vegetative, reproductive, and cellular growth and development, previously [50,51].

## 3. Proteomic Analysis of Pollen-Pistil Interaction in SI Response

If one plant is hermaphrodite, both stamen and pistil from the same flower, it has more chance to self-fertilize. Self-fertilization means that the offspring is obtained within a short time and is identical genetically to the parent plant, which is in favor of maintaining stability with low genetic diversity. However, the genetic stability makes it difficult to adapt to the variable environments so that species can achieve optimized continuation. Therefore, it naturally led to the generation of cross-fertilization and evolution of various mechanisms for preventing self-fertilization [52]. To enhance diversity and obtain more chances to survive in such a changeable and complex world, the evolution of several morphologic and genetic barriers of self-fertilization has occurred in the life history of plants. For instance, dichogamy, namely the condition of anthers and pistils maturing at different times, can prevent self-fertilization. Except physical and suited isolation, discrimination between genetically related (self) and unrelated (non-self) pollen grains that then inhibit the related grains is one major genetic mode. That is self-incompatibility (SI), the failure of fertilization between the pollen and pistil from the same flower to produce zygote and endosperm [31]. To date, more than half of species in angiosperms have self-sterility and SI. 

Dissecting the mechanism of the barrier system contributes to understand the series of events for pollen-pistil interaction. In genetics, SI is controlled by a single S (Sterility) locus with high polymorphism, which encodes proteins in pistil and pollen, respectively, as the basis of recognition and reaction during reproduction. There are two principal genetic forms of SI, gametophytic (GSI) and sporophytic (SSI), which are distinguished by the decider for the phenotype of S-locus in pollen [53]. The representative family for SSI is Brassicaceae, while Solanaceae and Papaceraceae are model plants for GSI. The mechanisms of the two categories are diverged with different controlled genes within a special signal pathway and regulatory network [54].

### 3.1. Proteomic Analysis of the Gametophytic Self-Incompatibility Response

In GSI, the S phenotype of the pollen is determined by its own haploid genome and the mechanism of GSI is mainly related to the inhibition of the growth of pollen tube. In addition there are two mechanisms of the GSI incompatibility system, where Solanaceae, Rosaceae and Plantaginaceae depend on the S-RNase-based rejection system and diversely Papaveraceae undergoes the S-glycoprotein mediated Ca^2+^ signaling system [55]. The female determinant of SI in Papaveraceae is PrsS (*P. rhoeas* style S) secreted by the stigma. When SI is trigged with the self-pollen, programmed cell death and pollen inhibition begin with increased concentration of Ca^2+^ [56,57]. Here, the review focuses on the S-RNase-based self-incompatibility. 

**Figure 1:**
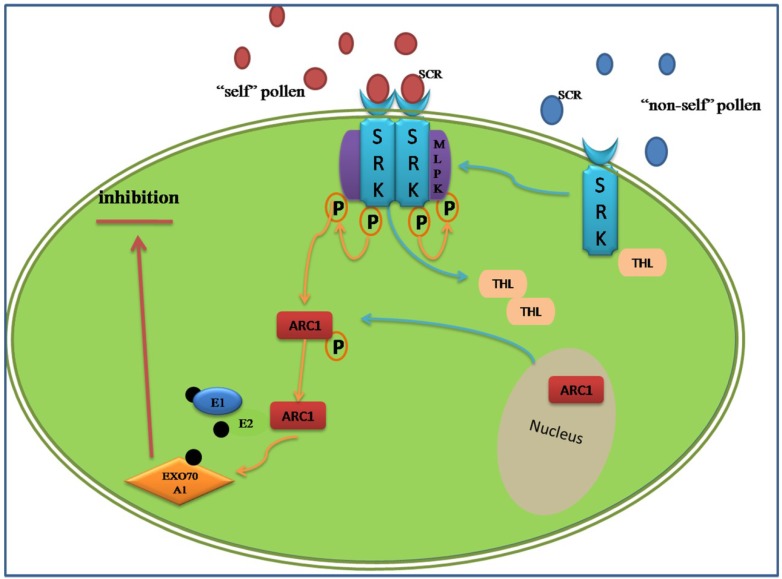
A simplified diagram of the S-RNase-based self-incompatible system. There was more than one type of S-locus F-box complex (SLF) to recognize all non-self S-Rnase. If the “self” S-RNases were compartmentalized in the vacuole for the degradation of HT-B, a protein indispensable to the release of S-RNase, the type of S-RNase may be cytotoxin contributing to RNA degradation during the extension of the pollen tube ending in the rejection of “self” pollen. On the contrary, the “non-self” S-RNase may be ubiquitinated thus degraded through the proteasomal pathway by the SLF complex.

The S-RNase-based rejection system is mediated through an interaction between S-locus ribonuclease (S-RNase, female determinant) and pollen tube-borne F-box proteins, S-locus F-box (SLF)/S-haplotype-specific F-box (SFB) (SLF/SFB male determinant) [58] (Figure 1).

The first S-specific proteins were found in *Nicotiana alata* in the stigma and style by isoelectric focusing [59]. Then, researchers confirmed that the S-specific proteins from different S-haplotypes in *Nicotiana alata* were ribonucleases with the RNase activity of inhibition of pollen tube growth in SI response [60].The similar approaches, including 2-D GE, were used in other Solanaceae and Rosaceae family to isolate and analysis of the style S-glycoprotein, such as *Petunia hybrida* [61,62] and Japanese pear [63,64]. The S-specific proteins were renamed S-RNases. The special and basic glycoproteins secreted into the extracellular matrix of the stigma, transmitting tract, and the inner epidermis of the ovary after pollination, which are cytotoxin contributing to RNA degradation during the extension of the pollen tube ending in the rejection of “self” pollen. The sequence analysis shows S-RNase has five conserved regions, C1 to C5, and two specially hyper-variable regions, HVa and HVb, which all contribute to the S-specific recognition [5]. However, there are not enough evidences to prove which domain determines the recognition.

To understand the mechanism of GSI, the male determinant related to S-RNase should be fully unfolded. The S-determinant should fulfill three rules as follows: (i) linkage to S-RNase gene; (ii) variable sequences comparing different S-haplotypes; (iii) expression in pollen, specifically. Based on the above characteristics, the male S-specific genes were surfaced. In *Antirrhinum*, *AhSLF-S2* was identified as a novel F-box gene closely involved in the interaction of SI, which is polymorphic and expressed in *tapetum*, microspores and pollen grains, specifically. The protein encoded by *AhSLF-S_2_* had a conserved F-box domain [65]. Similarly, SLF was shown to be the pollen S-determinant in *Petunia inflate* and *Prunus mume* [66,67]. Further studies predicted that Pi SLF was one component of an E3 ligase complex, cooperating with PI CUL1-G and SBP1, to mediate ubiquitination of non-self S-RNases, which may be degraded by the 26s proteasome [68]. The structure of SLF also confirmed the result with an ubiquitin-binding domain (UBD) in C-terminal region [69]. The transgenic functional assay was used to examine the interaction between the S_2_-SLF of *P. inflate* and non-self S-RNase, which proved that S_2_-SLF interacted with S_7_-RNase and S_13_-RNase but not with S_5_-RNase and S_11_-RNase. Through microRNA expression assays, it was also confirmed that there was more than one type of SLF protein to recognize all non-self S-RNase, of which each type of SLF would interact with a subset of non-self S-RNase [70].

In addition to inhibition and rejection, there is a theory of “compartmentalization”, whereby S-RNaseis compartmentalized in the vacuole for the degradation of HT-B, a protein indispensable to the release of S-RNase, and eventually promotes the compatible pollination [71]. Except the S-specific determinants, there are some modifier factors during GSI. The SBP1 and SCF-like E3 pitching in pollen could form a complex with SLF to allow the degradation or ubiquitination of non-self S-RNase, while the 120 kD a glycoprotein, the pistil extension-like protein III (PELPIII) and the transmitting tract-specific glycoprotein (TTS) are the S-RNase binding proteins in the pistil [52]. eEF1A, the instigator of the actin binding activity, could bind to S-RNase during SI in *Solanum chacoense* [72]. The exhaustive network of S-specific pollen rejection based on the S-RNases has been demonstrated in the reviews of McClure [71,73].

Actin-7, actin-8 and fructose bisphosphate aldolase-like protein were found only in self-pollinated pistil in apricots when compared with the across-pollinated by 2D-GE along with MS/MS analysis [74]. In apricots, the comparison between SC and SI cultivars at protein level showed there were 15 different proteins in two kinds of pistils, of which nine proteins were detected only in the SI pistils, including actin-7, a putative serine/threonine kinase and an S-RNase [75]. A protein expression profile in different stages of styles in “Hyuganatsu” (*Citrus*) was generated by 2D gel electrophoresis and MALDI-TOF/MS. The three stages were distinguished by location of the tube in the style after self-pollination: the tube was at the top of the styles in 1 and 3 days before anthesis (DBA) while it had reached the bottom of the styles in five DBA. There were 138 different protein spots among the three stages, in which 17 up-regulated and 26 down-regulated proteins were identified. As the plants were self-incompatible, the nine up-regulated proteins based on the pattern of 1 DBA > 3 DBA > 5 DBA may contribute to the transmission from SC to SI, which were related to SI response. Via Blast P analysis, there were nine proteins involved in tubulin alpha-4 chain, including probable rhamnose biosynthetic enzyme 1, 2,3-bisphosphoglycerate-independent phosphoglycerate mutase 1, fructokinase-2, allene oxide synthase, chloroplastic, luminal-bingding protein, photosystem 1 assembly protein. The first eight all participated in different biological processes while the last PG may be related to the SI reaction. The comparative studies in proteomics may be helpful in isolating the key proteins related to SI and eventually in explaining the reproduction process [76]. These studies uncover the special proteins in SC and/or SI process by proteomic approaches, which may also expand our insights on the possible pathways related to the interactions during both compatible and incompatible pollination. 

### 3.2. Proteomic Analysis of the Sporophytic Self-Incompatibility Response

Sporophytic self-incompatibility (SSI) is triggered by the interaction between a polymorphic stigma receptor and its pollen ligand, if the expressed genotypes of S allele are alike. The restriction ends in the failure of the development of the pollen tube. 

**Figure 2:**
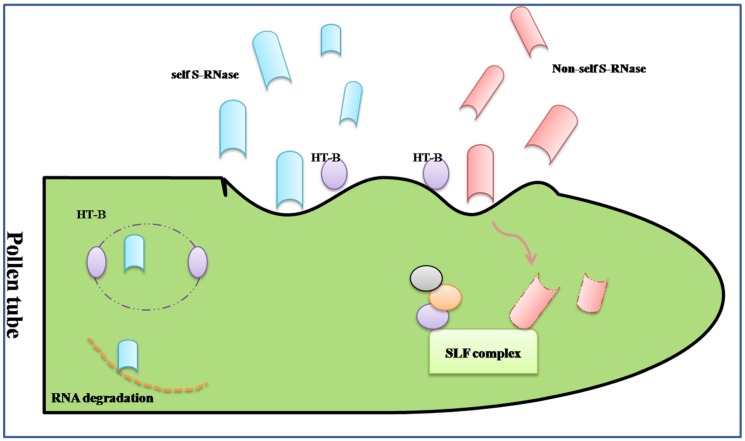
Amode of sporophytic self-incompatibility signaling pathway regulated by the ARCI. When the cognate S-locus cysteine-rich protein (SCR) binds to the extracellular domain of SRK, its intracellular domain is activated and relieves inhibition from THL proteins. A phosphorylation cascades transfer from the MLPK to ARC1. The phosphorylated ARC1 E3 ubiquitin ligase, supported by the E1 and E2 ligase, may activate the ubiquitination and degradation of EXO70A1. The ARC1-mediated degradation of EXO70A1 leads to the inhibition secretion of “compatible” factors to reject the “self” pollens.

In Brassicaceae, the genetic factors encoded by *S-locus* are S-locus receptor kinase (SRK), which is a transmembrane Ser/Thr receptor kinase on the epidermis of the stigma papilla cells and functions as the female S-determinant, and S-locus protein 11 (SP11, or S-locus cysteine-rich protein, SCR). Its expression level in pollen is as high as that in the male determinant. The interaction of SRKn–SCRn results in the compatibility or incompatibility based on the polymorphism of the two proteins [77]. Even if the intracellular signal pathway is still not acquainted entirely, a model of ubiquitin-proteasomal degradation based on the interaction between armadillo-repeat-containing 1 (ARC1) and EXO70A1, a substrate of ARC1, has been demonstrated (Figure 2) [78,79]. The degradation of these proteins may lead to the generation of SI, in other words, the predicted proteins represent various compatibility factors required for pollen germination and growth [80]. Around the SI pathway, the positive regulator M locus protein kinase (MLPK), which can recognize and phosphorylate ARC1, and the negative regulators THL1 and THL2, which belong to the thioredoxin-h family, were all discovered participating in the incompatibility response in Brassicaceae [81,82]. 

Based on the above, an accurate view of alternation in abundance of proteins within SI is a way to understand pollination and fertilization, comparing the similar but direct studies on pollen and/or pistil. The change of proteins can either be up-regulated or down-regulated. The purpose of this research was to obtain the comparative protein profile of SI reproductive tissues, and the differentially expressed proteins were predicted by functional classification, which may provide the mechanism of SI system. To explore this point, a changeable protein list on SI response was acquired by 2D-DIGE and mass spectrometry. Nineteen decreased proteins were identified and predicted to be involved in various pathways including biosynthetic pathways, signaling transduction pathway and cytoskeletal organization. Based on GO annotation, the 19 protein candidates were classified into six groups: metabolism (SLR glycoprotein, RUBISCO and so on), trafficking (annexin), development (actin), translation (GTP and several putative chloroplast translation), chaperone (protein disulphide isomerase), and structural (alpha 2–4 tubulin). Analyzing the various functions of these proteins can help to understand their mechanisms for down-regulation, consistent with the SI response. Compatibility between pharmacology and cell biological techniques—including using mutant lines and depolymerization of alpha 2–4 tubulin and microtubule (MT) in the stigmatic papilla contribution to the process of compatible pollen acceptance, which is likely mediated by EXO70A1—has been shown. Combing the down-regulated tubulin in SI, it indicates that the alteration of MT dynamics cannot affect the SI response [83]. Similar selection of candidate proteins involved in the SI system were reported in non-heading Chinese cabbage, of which traces of pistils were found at 0 h and 2 h after pollination in SI and SC lines. Among 22 potential proteins, the UDP-sugar pyrophosphorylase (USPase) and DNADP-dependent glyceraldehyde-3-phosphate dehydrogenase, two proteins classified in the energy metabolism, were down-regulated at 2 h after incompatible pollination, which showed the existence of sucrose degradation and ATP supply during the SI response. Similar analysis in the up-regulated methionine synthase (METS), involving the protein methylation, implied that DNA methyltransferases might have a role in SI response, which is related to post-translational modification in SI [84]. Contrary to incompatible pollen germination, the dynamics of proteins’ file established in *Brassica napus* showed that the enzymes involved in glycolysis, TCA cycle and electron transport chain were up-regulated in germinating pollen compared to the mature pollen, such as glyceraldehydes-3-phosphate dehydrogenase, malate dehydrogenase and cytochrome b5 reductases [24]. This indicated that the circulation of energy materials was necessary during normal pollination, while the down-regulated proteins in SI may provide opportunity to reject and suppress the “self” pollen. 

## 4. Conclusions and Perspectives

Proteomics studies on self-compatible and self-incompatible responses have immensely contributed to our understanding of the interaction between pollens and pistils. The analysis of the SI system, a phenomenon exhibiting in numerous plant species, gives a new insight in uncovering the mechanisms. The protein and mRNA level can be explained in the transcriptional and translational levels. The two fields are mutually reinforcing. The transcriptional program obtained by a comparison between 0–30 min stigmas of *Brassica napus* following the SI and compatible pollinations were used to uncover the genes participating in the compatible and incompatible responses via microarrays. One of the results showed that the absence of un-regulated genes in SI response was consistent with the down-regulated proteins following SI. This study’s results are consistent with the findings of previous study [85]. However, 33% of genes experienced different variation trends in mRNA levels compared with the protein levels in the pistils of non-heading Chinese cabbage [84]. The difference between the two levels may result from post-transcriptional regulation or post-translational modification [83]. Post-translational modifications include glycosylation, phosphorylation and ubiquitination [7]. So far, these modifications were identified in the reproductive process, involving the protein interactions, signaling transductions, protein degradations, and so on. For example, the SRK would be auto-phosphorylated for the homoplastic SCR from the pollen in Brassica. The phosphorylation cascades pass on to MLPK to transmit incompatible signals, which can activate ARC1, holding the E3 ligase, and leading to the ubiquitination and degradation of protein substrates [79,82]. In addition to the post-translational modification, the interaction partners and the sub-cellular localization also make sure that the transcript levels cannot reveal the dynamic protein properties. So, to remedy the imperfection of transcriptomics, the dynamics of proteins involved in the pollen and/or pistil (pre-pollination and after-pollination) should be tested at multiple time-points based on the development of MS technologies, which can be used by virtue of the peptide sequencing and isotope labeling methods to play a great role in obtaining the dynamic profile during the interaction between pollens and pistils. 

However, the coverage of proteins during the SI and SC processes is limited for different reasons, such as the protein expressions in space-time effect and the low-abundance of proteins. To overcome these limitations, multiple, more sensitive technologies should be developed. Therein, multi-dimensional protein identification technology (Mud PIT) can help to capture the proteins at multiple time-points [6]. Besides, the proteomics screen the candidates especially, which requires verifying their special functions in the pollen-pistil interaction via various molecular and genetic methods. Given the difficulties in the collection of reproductive tissues, the multidisciplinary fields and approaches within transcriptomics, proteomics and metabolomics should be combined to reveal the global network involved in the process of pollination. 
